# Heterologous expression of 1,4-benzoquinone reductases from white-rot fungi in *Saccharomyces cerevisiae* confers protection against *p*-benzoquinone toxicity

**DOI:** 10.1186/s12934-026-03001-1

**Published:** 2026-04-08

**Authors:** Mateo Bello-Villarino, Leif J. Jönsson

**Affiliations:** Department of Chemistry, Umeå University, SE-901 87 Umeå, Sweden

**Keywords:** 1,4-Benzoquinone reductase, *p*-Benzoquinone, Lignin, Bioethanol, Inhibitor, *Saccharomyces cerevisiae*, *Trametes versicolor*, *Phanerochaete chrysosporium*

## Abstract

**Background:**

Ethanolic fermentation by *Saccharomyces cerevisiae* is a key part of biochemical conversion of lignocellulosic feedstocks. Although abundant, lignocellulose typically requires pretreatment for efficient bioconversion. During pretreatment, inhibitory compounds are formed. *p*-Benzoquinone (*p*-BQ) is a lignin-derived inhibitor that is highly toxic to *S. cerevisiae*. White-rot fungi, efficient degraders of lignin, produce 1,4-benzoquinone reductases (QRDs), which have been suggested to play a role in defense against oxidative stress caused by quinones formed during lignin biodegradation. The hypothesis that QRDs from white-rot fungi can be used to engineer *S. cerevisiae* with improved resistance against quinone toxicity was explored in experiments with QRD-encoding genes, with and without an *N*-terminal putative leader sequence, the function of which has not yet been deciphered.

**Results:**

Constitutive recombinant expression of QRDs from the white-rot fungi *Trametes versicolor* and *Phanerochaete chrysosporium* in *S. cerevisiae* resulted in increased tolerance against *p*-BQ toxicity compared to an empty vector control containing insert-less plasmid, with QRD-expressing constructs requiring 34 to 42% less time to reach half-time of maximum growth in *p*-BQ conditions adverse for the growth of the empty vector control (10 mg L^− 1^). All QRD constructs were able to overcome *p*-BQ toxicity in concentrations that would otherwise be strongly inhibitory (20 mg L^− 1^). Full-length constructs of both *T. versicolor* and *P. chrysosporium* QRD typically exhibited 1.5 times higher specific activity than leaderless counterparts and were better detoxifiers. An inverse correlation between QRD enzymatic activity and the time necessary to reach half-time of maximum growth (R^2^ = 0.954) was observed during strongly inhibitory conditions. Transformants expressing QRD exhibited maintained glycerol and ethanol yields with increasing *p*-BQ concentrations, up to 0.44 g g^− 1^ ethanol at 10 mg L^− 1^ *p*-BQ, whereas the empty vector control exhibited increased glycerol yield and decreased ethanol yield, amounting to 0.15 g g^− 1^ ethanol at 10 mg L^− 1^ *p*-BQ.

**Conclusions:**

Heterologous expression of QRDs from white-rot fungi in *S. cerevisiae* confers protection against *p*-BQ toxicity. This investigation supports a role of QRD in the protection against toxic quinones and proposes a biodetoxification strategy against lignin-derived inhibitory *p*-BQ which can occur simultaneously with fermentation of sugars into ethanol in *S. cerevisiae*.

**Supplementary Information:**

The online version contains supplementary material available at 10.1186/s12934-026-03001-1.

## Background

The yeast *Saccharomyces cerevisiae* is a key player in biorefining based on biochemical conversion, which involves the conversion of biomass into bio-derived platform chemicals and added-value products [1–3]. This is due to the deep knowledge available regarding the cellular biology and molecular mechanisms of this GRAS microorganism, as well as its extensive toolbox for genetic modification and general tolerance against harsh conditions used in industrial contexts. *S. cerevisiae* is one of the most frequently used microorganisms in biotechnology [4] and is of interest not only for production of ethanol, but also for engineering for production of commodities such as 2,3‑butanediol [5] and PHB (polyhydroxybutyrate) [6].

In order for lignocellulosic biomass to be converted into bio-derived products, such as bioethanol, it needs to be pretreated to disrupt and solubilize its main components (cellulose, hemicelluloses and lignin) and hydrolyzed to release sugars that can finally be fermented by microorganisms [3, 7]. These processes also generate chemical species that act as inhibitors for fermenting microorganisms. The origin of some of these inhibitors, such as furan aldehydes and aliphatic carboxylic acids, has been traced back to hemicelluloses and cellulose, but compounds derived from the degradation of lignin, such as phenols and quinones, have also been shown to cause toxicity in *S. cerevisiae* when fermenting sugars into ethanol [8, 9]. The lack of effective molecular machinery to protect itself against inhibitors is consistent with the fact that environments where lignocellulosic degradation occurs are not specific ecological niches for *S. cerevisiae* [10, 11], as opposed to white-rot fungi.

Quinones are a class of compounds containing conjugated cyclic rings and ketone groups, considered oxidized derivatives of aromatic compounds. *p*-Benzoquinone (*p*-BQ) is a six-membered cyclic conjugated diketone. Whereas the corresponding phenols, i.e., hydroquinones such as 2,6-DMHQ [12], MHQ [12], and HQ [13, 14], have been known to be present in lignocellulosic hydrolysates for a long time, benzoquinones, such as 2,6-DMBQ and *p*-BQ, were more recently found to be ubiquitous in pretreated lignocellulosic biomass [15]. The finding was significant, as benzoquinones are much more toxic than hydroquinones. While *p*-BQ was found to completely inhibit cell growth, glucose consumption, and ethanol production at 20 mg L^− 1^, HQ exhibited no toxic effect at 20 and 200 mg L^− 1^, and had only minor negative effects at 1 g L^− 1^, which was the highest concentration tested [16]. Lower ranges of *p*-BQ concentrations, down to 2 mg L^− 1^, were also shown to have had at least modest inhibitory effects [15]. However, certain *S. cerevisiae* strains were found to have a limited resistance against *p*-BQ toxicity when the initial inoculation of microorganisms into the culture medium was as high as 10% (v/v) [17]. Regarding HQ, recent findings indicate that it can even have a detoxification effect when added to inhibitory lignocellulosic hydrolysates [18].

Basidiomycete fungi causing white-rot decay of wood such as *Phanerochaete chrysosporium* and *Trametes versicolor* are known for their capacity to mineralize lignin, i.e., degrade lignin all the way from the polymer to carbon dioxide. The enzyme 1,4-benzoquinone reductase (QRD) from *P. chrysosporium* was detected during the ligninolytic phase of fungal growth, suggesting a role in reduction of quinones produced during lignin degradation [19]. Given the potential of *p*-BQ to act as a redox-active toxin by undergoing a one-electron reduction to the semiquinone radical, which can be oxidized by molecular oxygen to generate super-oxide anions and ultimately highly toxic hydroxyl radicals derived from hydrogen peroxide, fungal quinone reduction has been hypothesized to serve as protection against quinone-derived oxidative stress [19].

Upon further studies of the expression regulation of the cDNA region encoding *P. chrysosporium* QRD, an apparent 71-amino-acid-residue leader sequence was identified before the experimentally determined *N*-terminus of the isolated corresponding protein [20]. This putative leader sequence aligned with the difference between the theoretical molecular mass of the translation product and the experimentally confirmed molecular mass of the QRD monomer, suggesting that the mature protein is the result of cleavage from a leader-containing precursor [20]. The cDNA encoding the QRD precursor contains four potential translation initiation sites, the first of which coincides with the first Met of the hypothetical leader sequence, and the second one corresponding to the last residue before the experimentally determined amino terminus of the protein.

There are no more studies in the literature regarding the function of the hypothetical leader sequence nor on the physiological role of QRDs in the metabolism of white-rot fungi. As H_2_O_2_ is not an inducer of QRD activity in fungal cultures, its function is more likely to be quinone-specific rather than a protection against oxidative stress in general [20]. Furthermore, expression of recombinant QRD from white-rot fungi has not been well investigated.

In order to explore the potential role of QRD in quinone biodetoxification (with potential downstream industrial applications in biochemical conversion of lignocellulosic biomass using yeast) and the function of the putative leader sequences present in the genes that encode them, QRDs from *T. versicolor* and *P. chrysosporium* were heterologously expressed in *S. cerevisiae*. This is to our knowledge the first instance of attempting to engineer resistance against quinones in the yeast *S. cerevisiae* by expression of recombinant QRDs from white-rot fungi.

## Methods

### Construction of *Saccharomyces cerevisiae* strains

*S. cerevisiae* BY4741 (MATa his3Δ1 leu2Δ0 met15Δ0 ura3Δ0) cells were used as the host organism to recombinantly produce 1,4-benzoquinone reductase (QRD) from the white-rot fungi *Trametes versicolor* and *Phanerochaete chrysosporium* using the vector p426 GPD (ATCC 87361), which constitutively expresses heterologous protein under the regulation of the GPD promoter. Genes encoding QRDs were ordered from Gene Oracle (Mountain View, CA, USA) and were cloned into p426 GPD in between restriction enzyme sites *Spe I* and *EcoR I*. The same CDS regions were used as a template for PCR amplification to obtain leaderless constructs of QRD lacking the first start codon. These leaderless constructs were designed to avoid the translation of putative leader peptides (the amino-terminal end of the QRD gene as described in [20] for *P. chrysosporium* and inferred from homology for *T. versicolor*) (see also Additional file 1: Fig. S1). The resulting amplicons were also cloned into p426 GPD in between the *Spe I* and *EcoR I* sites.

*S. cerevisiae* cells were made electrocompetent following protocol no. 4308 915.531 (Eppendorf, Hamburg, Germany) and the four constructs plus the p426 GPD vector without insert (for use as an empty vector control) were transformed into the host cells by electroporation using a BioRad (Hercules, CA, USA) Gene Pulser. Newly transformed cells were recuperated in 1 M sorbitol and immediately plated onto SD-Ura (2×Leu) solid medium supplemented with 1 M sorbitol, incubating them at 30 °C. Individual colonies were picked and grown to A_600_ of ≈ 1.0 for storage at -80 °C in 0.5 mL aliquots with 30% (v/v) sterile glycerol as cryoprotectant.

### Cultivation of *S. cerevisiae* constructs in *p*-benzoquinone-containing medium

Glycerol stocks were thawed and plated onto SD-Ura (2×Leu) agar plates. A single colony from each type of transformant was picked and transferred into 5 mL SD-Ura (2×Leu) liquid medium to be used as preinoculates, which were incubated at 30 °C, 150 rpm until an A_600_ of ≈ 1.0. Samples from the preinoculates were used to verify successful transformation through colony PCRs using the primers designed for construct development.

Cultures comprised of 50 mL SD-Ura (2×Leu) liquid medium were inoculated with 0.5% (v/v) of the corresponding preinoculate and supplemented with increasing *p*-BQ concentrations (0, 2, 10 and 20 mg L^− 1^) in triplicate. Cultures were incubated in 250 mL baffled flasks with vented caps at 30 °C, 150 rpm. Yeast growth was assessed through A_600_ measurements by diluting 100 µL of culture in 900 µL of sterile medium at different timepoints. Samples were taken at early (A_600_ of ≈ 1.0) and late (A_600_ of ≈ 4.0) logarithmic phase of growth. Biomass was separated from culture medium by centrifugation at 6000 × *g*, 4 °C for 15 min. The pellets were weighed and stored at -20 °C until further processing. The extracellular medium (ECM) was passed through a syringe filter with a 0.20 μm pore size and frozen at -20 °C until further processing.

### Enzymatic specific activity measurements

Samples taken from late log phase of growth were used to quantify protein content through Pierce BCA protein assay using BSA as a standard and to assess the presence of active QRD both spectrophotometrically and through activity staining after native gel electrophoresis. Biomass-containing pellets were thawed and resuspended in sodium citrate buffer pH 5.2, 50 mM. Glass beads (450–600 micron) were used to lyse the cells by vortexing in 15 s pulses intercalated with 15 s ice incubation pauses for a total vortex time of 1 min. Lysed biomass was centrifuged at 6000 × *g*, 4 °C for 5 min to separate soluble proteins in the intracellular medium (supernatant) from cell debris (pellet).

Spectrophotometric reaction mixtures (1 mL) contained 0.07 mM *p*-BQ and 0.10 mM of either NADH or NADPH buffered at pH 5.2 in sodium citrate buffer, 50 mM. The reaction was started by adding 50 µL of concentrated extracellular medium or intracellular extract and activity was measured by monitoring NAD(P)H oxidation at 340 nm in room temperature (~ 23 °C). An autoreaction substituting the enzyme sample for buffer was carried out to assess the spontaneous oxidation of NAD(P)H by *p*-BQ in the absence of enzyme. A substrate-less reaction was run substituting *p*-BQ for buffer. One unit (U) of enzymatic activity is defined as the amount of enzyme necessary to catalyze the oxidation of 1 µmol of NAD(P)H concomitantly with the reduction of 1 µmol of *p*-BQ per min. Enzyme activity was tested in the presence of either NADH or NADPH as an electron donor to assess donor preference. Native gels were run for activity staining using the conversion of MTT tetrazolium (yellow) to MTT formazan (blue-violet) by hydroquinones generated due to reductase activity. After running samples obtained from all constructs when cultured in the presence of 10 mg L^− 1^ *p*-BQ at a normalized concentration of 50 ng of protein per lane of the native gel in non-denaturing conditions, it was soaked in 50 mL of a solution containing 0.05 mM *p*-BQ, 1 mM NADH or NADPH and 2 mM MMT tetrazolium buffered at pH 8.0 in Tris-HCl buffer, 25 mM. An image of the zymogram was taken 30 min after the start of the incubation at RT.

### Measurements of glucose, ethanol and glycerol in extracellular medium

Liquid ECM samples were thawed, diluted 1:1 with ultrapure water and passed through a filter of 0.20 μm pore size into glass vials. The concentrations of glucose, ethanol, and glycerol were determined using an UltiMate 3000 HPLC instrument (Thermo Fisher Scientific, Waltham, MA, USA) equipped with a refractive index detector (RID). The separation column was Aminex HPX-87 H (Bio-Rad), the mobile phase was an aqueous solution of 0.005 M H_2_SO_4_, and the flow rate was 0.6 mL min^− 1^. The temperature of the column oven and the detector was 55 °C. For quantitation, an external calibration curve ranging from 0.05 to 25 g L^− 1^ was used. The Chromeleon 7.1 software (Thermo Fisher Scientific) was used for data analysis.

### Bioinformatic analysis of 1,4-benzoquinone reductases from white-rot fungi

A collection of hypothetical QRDs was obtained by searching for 1,4-benzoquinone reductase in UniProt and restricting the search with respect to taxonomy to category 5303 (Polyporales). The set of protein sequences obtained was assigned as 1,4-benzoquinone reductase in this database based on sequence similarity to a flavodoxin-like domain. The mRNA corresponding to each entry (Additional file 1: Table S1) was analyzed for start codons to evaluate the strength of their translation initiation in terms of similarity to the Kozak sequence 5’-(gcc)gccRccAUGG-3’. The corresponding amino-acid sequences of the hypothetical leader peptides and the hypothetical mature enzymes together with a reported NAD(P)H quinone oxidoreductase from *S. cerevisiae* were used to generate a dendrogram showing sequence similarity. Amino-acid sequences of QRDs from Polyporales fungi were aligned to assess homology using the align tool from the UniProt Consortium.

## Results

### Growth in medium with *p*-benzoquinone

**Fig. 1:**
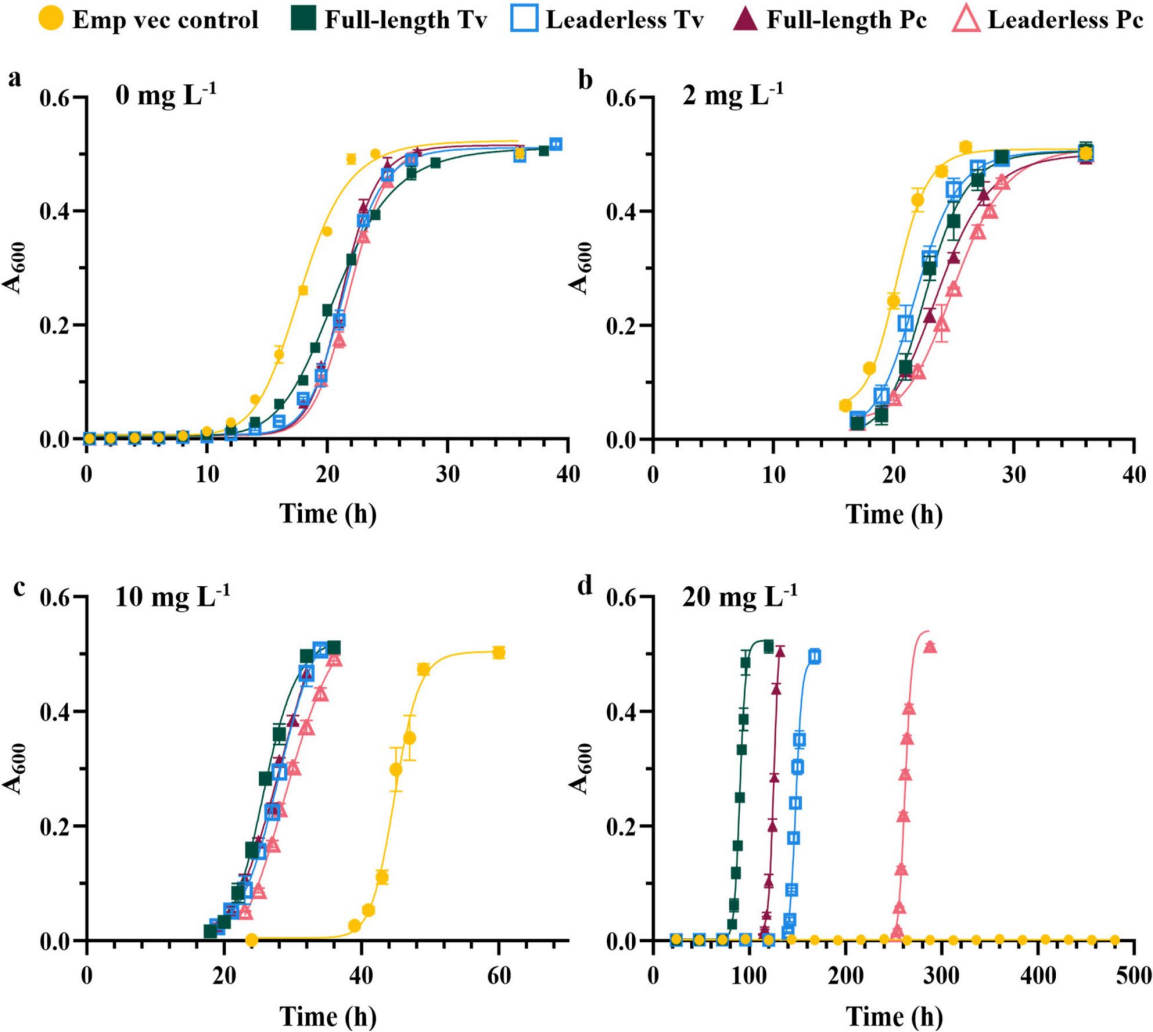
Growth curves of *S. cerevisiae* transformants with QRD-expressing constructs and empty vector control (Emp vec control) in cultures with different *p*-BQ concentrations. A_600_ values correspond to 10 × dilutions. Initial *p*-BQ concentrations (mg L^− 1^) in culture medium: **a** 0; **b** 2; **c** 10; **d** 20

When cultured in the absence of *p*-BQ, recombinant expression of QRDs showed an unfavorable effect on the growth of *S. cerevisiae* compared to the empty vector control (Fig. 1a), on average taking 1.2 times longer to reach the same growth. Specifically, the empty vector control had a half-time growth of around 18 h, whereas the QRD constructs had a half-time growth of 21–22 h and showed a reduced specific growth rate (Table 1) compared to the empty vector control. With 2 mg L^− 1^ *p*-BQ (Fig. 1b), the half-time growth increased to 20 h for the empty vector control and to 22–25 h for the QRD constructs, while the specific growth rate differences in between the empty vector control and the QRD constructs were not as notable as when no *p*-BQ was present in the culture medium (Table 1). Both without and with 2 mg L^− 1^ *p*-BQ, the leaderless PcQRD construct exhibited the longest time for half-way growth (Fig. 1; Table 1).

**Table 1:** Half-time growth (HTG) and specific growth rate (µ) of yeast transformants with QRD-expressing constructs and empty vector control

Initial [*p*-BQ]	0 mg L^− 1^		2 mg L^− 1^		10 mg L^− 1^		20 mg L^− 1^	
	HTG	µ	HTG	µ	HTG	µ	HTG	µ
Empty vector control	17.9	0.42	20.3	0.37	44.8	0.40	N/A	N/A
Full-length TvQRD	20.8	0.33	22.7	0.38	25.9	0.39	90.2	0.35
Leaderless TvQRD	21.4	0.36	22.0	0.37	28.4	0.37	148.4	0.39
Full-length PcQRD	21.3	0.38	23.6	0.37	29.5	0.36	125.6	0.37
Leaderless PcQRD	21.9	0.36	25.1	0.33	29.6	0.32	261.2	0.41

Values for HTG (in h) were calculated from the sigmoidal equation of the corresponding growth curve for each experimental condition and correspond to the time required to achieve half the maximum A_600_. Values for µ (in h^− 1^) were calculated from the logarithmic region of datasets

When the culture medium contained 10 and 20 mg L^− 1^ *p*-BQ, the growth of the empty vector control was strongly negatively affected (Fig. 1c) and completely impaired (Fig. 1d), respectively. With 10 mg L^− 1^ *p*-BQ, the half-way growth was around 45 h, 2.5 times longer than in medium without *p*-BQ (Table 1). In contrast, the QRD-expressing constructs were less affected by 10 mg L^− 1^ *p*-BQ (Fig. 1c) and exhibited growth even with 20 mg L^− 1^ *p*-BQ (Fig. 1d). With 10 mg L^− 1^ *p*-BQ, the half-way growth of the QRD constructs was 26–30 h, 1.2–1.4 times longer than for medium without *p*-BQ and 34 to 42% lower than the time required for the empty vector control. Leaderless constructs exhibited slightly longer half-way growth than full-length constructs, TvQRD constructs exhibited shorter half-way growth than PcQRD constructs, and, as before, leaderless PcQRD exhibited the longest half-way growth among the QRD constructs (Table 1). With 20 mg L^− 1^ *p*-BQ, the difference between the constructs became even clearer: leaderless constructs exhibited longer half-way growth than full-length constructs, TvQRD constructs exhibited shorter half-way growth than PcQRD constructs, and, among all QRD-expressing constructs, leaderless PcQRD exhibited the longest half-way growth. Specifically, the construct expressing full-length TvQRD reached half of maximum growth 1.4 times faster than the next-best construct, full-length PcQRD. The leaderless TvQRD construct took 1.6 times longer than its corresponding full-length construct to reach half of maximum growth, and leaderless PcQRD required over twice as long time as its full-length counterpart and almost three times longer than full-length TvQRD. Thus, the results consistently demonstrate that transformants with full-length constructs were better detoxifiers than their leaderless counterparts, and that transformants with the QRD from *T. versicolor* performed better than those with the QRD from *P. chrysosporium*.

### Enzymatic activity in QRD-expressing constructs

**Fig. 2:**
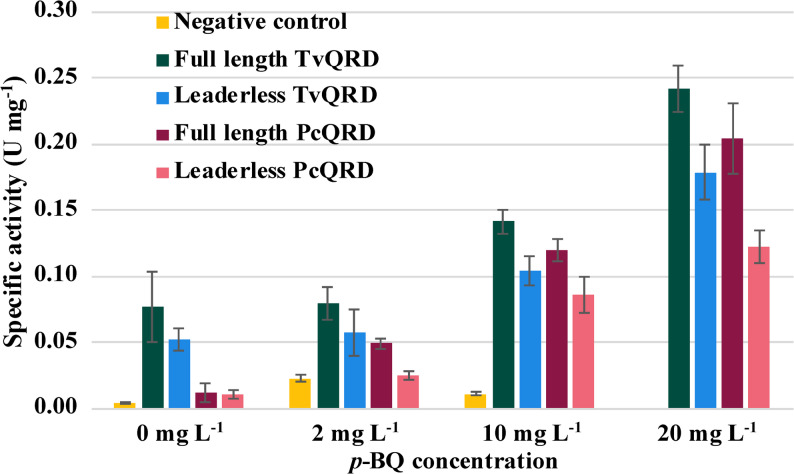
Specific activity measurements expressed in units of QRD activity (U) per mg of protein. Error bars represent the standard deviation of triplicates. Due to its lack of growth, there is no data available for the empty vector control cultivated in medium with 20 mg L^− 1^ *p*-BQ

**Fig. 3:**
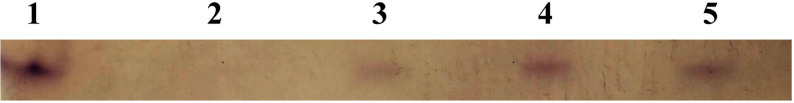
Zymogram of a native PAGE showing samples of intracellular medium from different constructs after growth (A_600_=4.0) in cultures supplemented with 10 mg L^− 1^ *p*-BQ: Lane 1, full-length TvQRD; lane 2, empty vector control; lane 3, leaderless PcQRD; lane 4, leaderless TvQRD; lane 5, full-length PcQRD

The recombinant expression of QRDs was verified through spectrophotometric enzyme activity assays (Fig. 2; Additional file 1: Table S2) and enzyme activity staining after native PAGE (Fig. 3; Additional file 1: Fig. S2). Enzymatic activity values took into account the slight decrease in absorbance observed in the autoreaction and reflect only the catalytic activity caused by each experimental condition. There was a low activity in the empty vector control (≤ 0.022 U mg^− 1^), except for cultures with 20 mg L^− 1^ *p*-BQ in which the empty vector control did not grow. The background activity can probably be related to endogenous QRD activity in *S. cerevisiae* [21], as substrate-less reactions showed no change in absorbance. Enzymatic activity was assessed in the presence of either NADH or NADPH as electron donors, which were added in the same concentration to the spectrophotometric reactions. There was no difference between the two, suggesting that both cofactors are equally efficient.

With or without *p*-BQ in the medium, full-length constructs always exhibited higher specific activity than the corresponding leaderless construct (Fig. 2): 1.4–1.5 times higher for TvQRD and 1.2-2.0 times higher for PcQRD. As the values for leaderless PcQRD with 0 or 2 mg L^− 1^ *p*-BQ were very low (≤ 0.025 U mg^− 1^) and close to those of the empty vector control (Fig. 2), the values obtained for PcQRD with 10 or 20 mg L^− 1^ *p*-BQ should be more reliable. With 10 mg L^− 1^ *p*-BQ, both full-length PcQRD and full-length TvQRD resulted in 1.4 times significantly higher (*p* < 0.05) specific activity than the leaderless counterpart. The corresponding number for cultures with 20 mg L^− 1^ *p*-BQ was 1.6. Thus, full-length constructs of both TvQRD and PcQRD typically exhibited ~ 1.5 times higher specific activity than leaderless counterparts.

With or without *p*-BQ in the medium, TvQRD constructs always exhibited higher specific activity than the corresponding PcQRD construct (Fig. 2). Ignoring very low values (≤ 0.025 U mg^− 1^) close to those of the empty vector control (≤ 0.022 U mg^− 1^), TvQRD constructs exhibited 1.2–1.6 times higher activity than their PcQRD counterparts.

For each of the QRD constructs, the specific activity always increased with increasing *p*-BQ concentrations in the medium (Fig. 2). Focusing on the TvQRD constructs, which exhibited specific activities that were always high above the background, the specific activity in medium with 2 mg L^− 1^ *p*-BQ was just a few percent above that in medium with no *p*-BQ, but with 10 mg L^− 1^ it was two times higher and with 20 mg L^− 1^ it was three times higher. There was an inverse correlation between QRD enzymatic activity and the time necessary to reach half-time of maximum growth (R^2^ = 0.954) in strongly inhibitory conditions (20 mg L^− 1^ *p*-BQ).

Visual inspection of native PAGE gels stained for activity detection using samples from all constructs at growth conditions supplemented with 10 mg L^− 1^ *p*-BQ showed band intensity results consistent with values obtained from spectrophotometric activity assays, i.e., full-length TvQRD exhibited the strongest signal and leaderless PcQRD the weakest. The zymogram depicted in Fig. 3 was taken 30 min after the start of incubation at RT with 2 mM MTT tetrazolium, 50 µM *p*-BQ and 1 mM NADH. The same experiment was repeated using NADPH as electron donor and results were similar.

Samples from conditions showing the lowest and highest signal (Fig. 3) were also run for comparison in denaturing conditions and stained with Coomassie Blue (Additional file 1: Fig. S3), but visual inspection was not enough to detect the presence of heterologously produced protein.

### Production of ethanol and glycerol

Table 2 shows the yields of ethanol and glycerol on consumed glucose. The empty vector control exhibited progressively decreasing ethanol yield and increasing glycerol yield until the highest *p*-BQ concentration that the transformant could tolerate (10 mg L^− 1^) was reached. Specifically, the ethanol yield significantly decreased (*p* < 0.01) from 0.40 (no *p*-BQ) to 0.15 g g^− 1^ (10 mg L^− 1^ *p*-BQ), and the glycerol yield significantly increased (*p* < 0.01) from 0.06 (no *p*-BQ) to 0.09 g g^− 1^ (10 mg L^− 1^ *p*-BQ). In contrast to the empty vector control, the transformants with the four QRD constructs did not exhibit any clear trends with regard to ethanol and glycerol yields with increasing *p*-BQ concentrations in the medium. There were minor differences between the transformants, but ethanol yields were in the range 0.34–0.44 g g^− 1^ and glycerol yields were in the range 0.03–0.07 g g^− 1^, even at 20 mg L^− 1^ *p*-BQ.

**Table 2:** Ethanol and glycerol yields of the empty vector control and QRD-expressing constructs

Initial [*p*-BQ]	0 mg L^− 1^		2 mg L^− 1^		10 mg L^− 1^		20 mg L^− 1^	
Analyte	Ethanol	Glycerol	Ethanol	Glycerol	Ethanol	Glycerol	Ethanol	Glycerol
Empty vector control	0.40 (0.01)	0.06 (0.00)	0.35 (0.02)	0.07 (0.00)	0.15 (0.02)	0.09 (0.00)	N/A	N/A
Full-length Tv	0.41 (0.01)	0.05 (0.00)	0.35 (0.02)	0.04 (0.00)	0.37 (0.03)	0.04 (0.00)	0.41 (0.01)	0.05 (0.00)
Leaderless Tv	0.40 (0.00)	0.07 (0.00)	0.36 (0.01)	0.04 (0.00)	0.44 (0.00)	0.05 (0.00)	0.42 (0.02)	0.07 (0.01)
Full-length Pc	0.36 (0.00)	0.05 (0.00)	0.35 (0.01)*	0.04 (0.01)	0.37 (0.05)	0.05 (0.00)	0.34 (0.01)*	0.03 (0.00)
Leaderless Pc	0.38 (0.01)	0.07 (0.00)	0.38 (0.00)	0.07 (0.00)	0.39 (0.00)	0.07 (0.00)	0.40 (0.00)	0.07 (0.00)

Values were obtained dividing the amount of produced ethanol or glycerol (g) by the amount of consumed glucose (g) in culture conditions with different concentrations of *p*-BQ at late logarithmic growth stage (A_600_=4.0). Values are averages of six replicates (technical duplicates for each one of biological triplicates), except those marked with an asterisk (*), which are averages of four replicates (technical duplicates for each one of biological duplicates)

### Bioinformatic analysis of 1,4-benzoquinone reductases from white-rot fungi

**Fig. 4:**
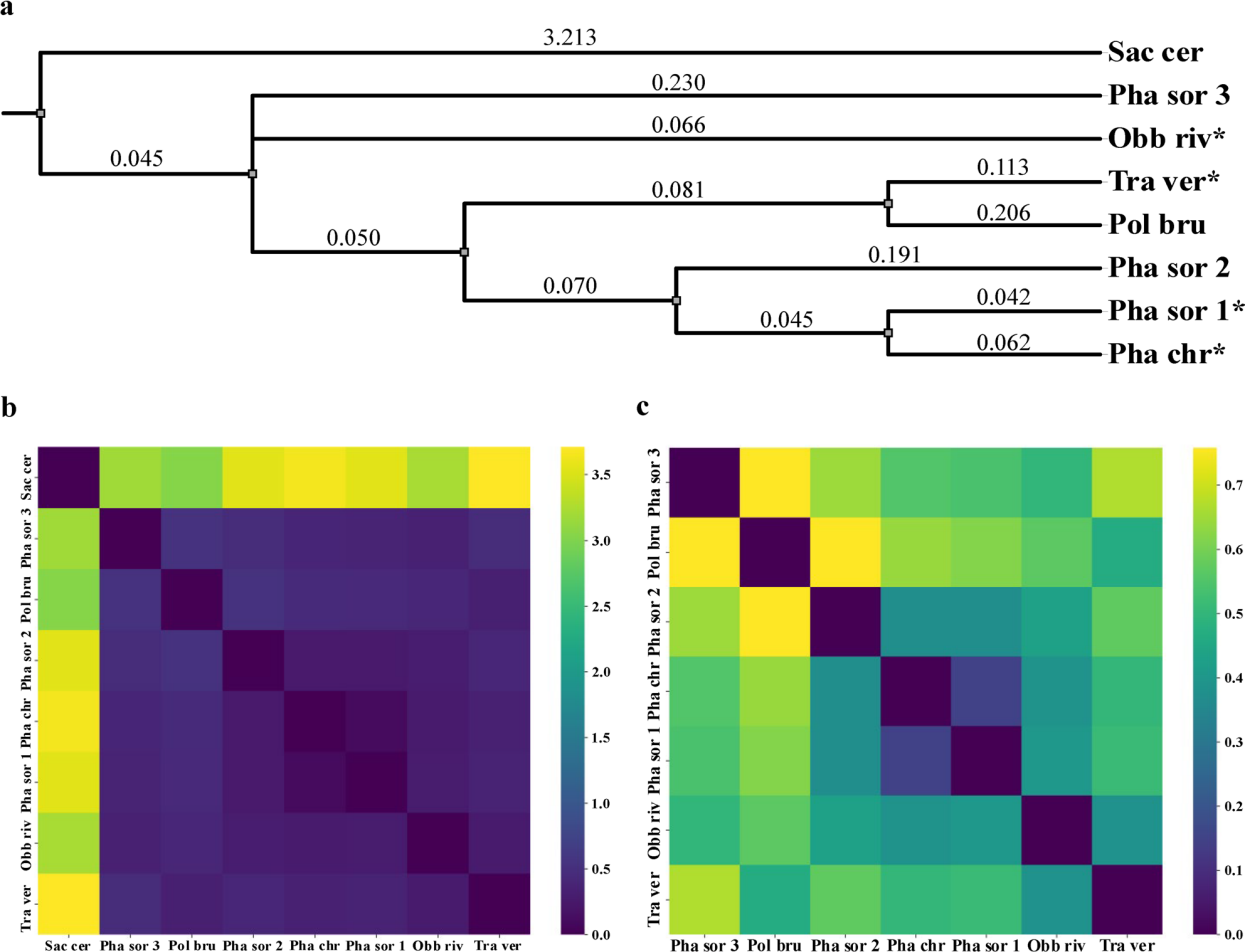
(a) Protein dendrogram of reported 1,4-benzoquinone reductases in genomes of Polyporales fungi and *Saccharomyces cerevisiae* (Sac cer). Values above each branch represent their distance to the most immediate node. Organisms included are *Polyporus brumalis* (Pol bru), *Trametes versicolor* (Tra ver), *Phanerochaete sordida* (Pha sor) with three potential QRDs, *Obba rivulosa* (Obb riv) and *Phanerochaete chrysosporium* (Pha chr). Abbreviations ending with an asterisk (*) indicate QRDs with an *N*-terminal putative leader peptide. (b) Heatmap of pairwise evolutionary distances between primary sequences of all analyzed QRDs. (c) Heatmap of pairwise evolutionary distances between primary sequences of QRDs from Polyporales fungi

Figure 4a shows a dendrogram depicting the distance in between amino-acid sequences of reported 1,4-benzoquinone reductases in genomes of Polyporales fungi, which includes the sequences of TvQRD and PcQRD studied in this investigation, as well as a reported NAD(P)H quinone oxidoreductase from *S. cerevisiae*. Of the seven Polyporales sequences compared, four contained a hypothetical leader sequence, and the other three did not. Even in the cases where no leader sequence is deemed to be present based on upstream analysis of the start codon, the analyzed nucleotides from the whole genome shotgun sequence contain both introns and exons. In the hypothetical case that an intron occurs in the middle of a sequence encoding a potential leader peptide, there is a risk of false negatives. The distance between the *S. cerevisiae* branch of the dendrogram and the cluster containing QRDs from Polyporales fungi (Fig. 4a) highlights the lack of homology between them, as exemplified in Fig. 4b and in contrast to the higher degree of similarity of the QRDs from white-rot fungi (Fig. 4c).

Sequences diverged in length, those containing a putative leader sequence having around 50 (*T. versicolor* and *Obba rivulosa*) or roughly 70 (*P. chrysosporium* and *Phanerochaete sordida* 1) more amino-acid residues than those that did not (*Polyporus brumalis*, and *P. sordida* 2 and 3), which were made up of approximately 200 residues (Additional file 1: Fig. S1). All potential mature translation products would have a predicted molecular mass ranging in between 21.2 and 21.4 kDa.

Generally, all sequences containing a hypothetical leader sequence showed a “stronger” (i.e., more closely resembling the consensus Kozak sequence) translation initiation site in the second potential place compared to the first one. It must be taken into account that: (*i*) the Kozak sequence forces a G immediately after the start codon, which restricts the second translated amino-acid residue to Val, Ala, Asp, Glu, or Gly, (*ii*) the amino-acid residue immediately after the start codon of the leader peptides is a Cys, and (*iii*) the amino-acid residue immediately after the start codon of the mature proteins is a Ser, a Pro, or (in one case) an Ala.

The first 15 amino-acid residues of the putative leader sequences show a high degree (66%) of identity (Additional file 1: Fig. S1). After the first 15 residues, identity percentage decreases. The length of the leader sequences differs greatly: 46 amino-acid residues for *O. rivulosa*, 54 for *T. versicolor* and 71 for *P. chrysosporium*. The remainder of the putative leader peptides do show an unusually high prevalence of prolines (27.1% for *P. chrysosporium*, 22.2% for *T. versicolor* and 17.4% for *O. rivulosa*).

## Discussion

### Growth in medium with *p*-benzoquinone in relation to QRD activity

Growth in medium with *p*-BQ concentrations ranging from 2 to 20 mg L^− 1^ was found to have an inhibitory effect on *S. cerevisiae* BY4741. The observation that a *p*-BQ concentration as low as 2 mg L^− 1^ had an inhibitory effect on *S. cerevisiae* agrees with a previous observation with another yeast strain (commercial bakers’ yeast) [15]. Pretreatment liquids from biomass pretreated using different methods were reported to contain *p*-BQ concentrations up to around 6 mg L^− 1^ [15], which highlights the likelihood of encountering concentrations in industrial contexts that are sufficiently high to cause inhibition. Furthermore, these concentrations are greatly dependent on the combined severity (residence time, temperature, and acidity) of the pretreatment and the liquid-to-solids ratio used in the pretreatment, stressing the importance of developing strategies for resistance against even higher concentrations.

Heterologous expression of white-rot QRDs in *S. cerevisiae* provided protection against inhibition derived from *p*-BQ supplementation, allowing constructs recombinantly producing the enzyme to overcome the toxic effects that proved inhibitory for growth of the empty vector control. The correlation between enzymatic activity and growth suggests that higher QRD enzymatic activity confers greater protection against *p*-BQ toxicity. It is a possibility that the slower growth of QRD-expressing transformants in medium without *p*-BQ, or with very low concentrations of *p*-BQ (such as 2 mg L^− 1^), is the result of a metabolic toll imposed by recombinant expression. The lack of preference in electron donor of PcQRD aligns with the previous report [20] and it strengthens the enzyme similarity with TvQRD.

The fact that the specific activity increased with increasing *p*-BQ concentration in the medium warrants an explanation, as the GPD promoter from the glyceraldehyde-3-phosphate dehydrogenase gene of *S. cerevisiae* is supposedly constitutive. It is a possibility that stress conditions caused by *p*-BQ affected transcription levels. There is also a possibility that the *p*-BQ levels affected the average copy number of the plasmid, as growth conditions, including medium composition, and stress could affect the average copy number. With no *p*-BQ in the medium, the plasmid and the expression of recombinant QRD would cause a metabolic burden on the cells. With increasing *p*-BQ concentration, the plasmid and expression of recombinant QRD would be a metabolic asset rather than a burden, something that could potentially favor cells with a high plasmid copy number.

### Comparison of QRD constructs

Full-length constructs seemed to offer greater protection against the detrimental effects of *p*-BQ than their leaderless counterparts. Although the explanation behind this phenomenon cannot be fully elucidated from this study, this finding is aligned with the only work in literature that included a study of the QRD leader [20]. The natively produced QRD from *P. chrysosporium* diverges in the molecular mass of its monomer by 32% when compared to the theoretical mass of the product encoded in its corresponding gene. Akileswaran et al. [20] attributed this to the cleavage of a putative *N*-terminal leader sequence comprised of 71 amino-acid residues, further supported by *N*-terminal sequencing of the purified polypeptide, although the possibility of translation initiation from the Met at position 71 was not discarded. The similarities in post-translational modification capabilities between *S. cerevisiae* and filamentous fungi could offer the yeast the same possibilities to post-translationally process the recombinant QRDs in the same way as their native hosts do. The putative leader sequence is also present in QRD sequences from basidiomycetes *P. brumalis*, *O. rivulosa*, and one of the three QRDs encoded in the genome of *P. sordida*, but absent in the other two and the QRD of *P. brumalis* (Fig. 4). The lower degree of similarity among these putative leader sequences compared to their catalytic portion as well as their intrinsically disordered character due to the overall lack of secondary structure in their predicted three-dimensional structure using the AlphaFold Server (data not shown) hints at them being low complexity regions [22]. Together with the increased protection against *p*-BQ found for full-length constructs, this opens the possibility of the leader possessing a functional benefit, e.g., improving production levels, improving folding of the three-dimensional structure, improving stability, or preventing premature enzyme activity.

The performance of QRD from *T. versicolor* regarding protection from *p*-BQ toxicity was generally better than that from *P. chrysosporium*. Although an intracellular QRD has been natively purified from *T. versicolor* and partially characterized [23], it had different size and different properties compared to *P. chrysosporium* QRD, and its amino-acid sequence was not reported, so it is not possible to ascribe the enzymatic properties detailed therein to the enzyme investigated in the present study. However, the reported molecular mass (41 kDa monomer for *T. versicolor* QRD versus a dimer composed of 22 kDa monomers for the *P. chrysosporium* QRD) and electron donor preference (NADH favored rather than NADPH in contrast to PcQRD, for which no preference was reported) suggest that the two enzymes from *T. versicolor* are not similar despite sharing a name.

### Metabolic performance

Despite the lack of clear trends regarding ethanol production in relation to *p*-BQ concentrations for experimental conditions, ethanolic fermentation occurred in all constructs, in contrast to the decreasing ethanol yield shown by the empty vector control (Table 2). Furthermore, the increased glycerol yield in the empty vector control as *p*-BQ supplementation increased could be interpreted as a response from the yeast to compensate a redox imbalance [11, 24]. The NAD^+^ that is required for glycolysis to proceed would typically be regenerated by reduction of acetaldehyde to ethanol by alcohol dehydrogenase. In case of redox imbalance, NAD^+^ can instead be regenerated through the glycerol shunt, in which dihydroxyacetone phosphate is reduced to glycerol-3-phosphate, which is then dephosphorylated to glycerol. This would also result in decreased ethanol yield, which agrees with what was observed in the experiments with the empty vector control. The heterologous expression of QRD prevented this from occurring and the ethanol yield was maintained. This indicates that recombinant QRD expression in *S. cerevisiae* is a viable strategy that could be further developed and incorporated into industrially relevant strains as a means of protection against *p*-BQ-derived cytotoxicity.

## Conclusions

This study proposes a biodetoxification strategy against lignin-derived inhibitor *p*-BQ which can occur simultaneously with fermentation of sugars into ethanol in the yeast *S. cerevisiae*, the preferred microorganism in industrial ethanol production and a common microbial host for development of production of other commodities. The validity of fungal 1,4-benzoquinone reductases as protection against quinone toxicity is shown both in decreased cultivation times, maintained ethanol production, and low by-product (glycerol) formation. Recombinant production of fungal QRDs from white-rots in *S. cerevisiae* confers protection against quinone toxicity, and this approach could be further developed for use in different contexts where microbial fermentation is impaired by *p*-BQ inhibition. Although the leader sequence of fungal QRD was not necessary for successful heterologous expression in *S. cerevisiae*, it was found to be beneficial. Higher concentrations of *p*-BQ in the medium resulted in higher specific QRD activity. These phenomena warrant further studies in the future.

## Supplementary Information

Below is the link to the electronic supplementary material.

Supplementary Material 1: Fig. S1. Amino-acid sequence alignments of reported 1,4-benzoquinone reductases. Fig. S2. Zymogram of a native PAGE showing samples of intracellular medium from different constructs after growth (A_600_=4.0) in cultures supplemented with 10 mg L^− 1^ *p*-BQ. Fig. S3. SDS-PAGE gel showing intracellular extracts of different colonies from the empty vector control and full-length TvQRD. Table S1. Accession numbers of gene-level and protein-level sequences of QRDs included in the study. Table S2. Specific activity measurements.

## Data Availability

All data generated or analyzed during this study are included in this published article.

## Abbreviations

*p*-BQ: *para*-Benzoquinone
QRD: 1,4-Benzoquinone reductase
GRAS: Generally regarded as safe
2,6-DMHQ: 2,6-Dimethoxyhydroquinone
2,6-DMBQ: 2,6-Dimethoxybenzoquinone
MHQ: Methoxyhydroquinone
HQ: Hydroquinone
CDS: Coding sequence
PCR: Polymerase chain reaction
ECM: Extracellular medium
BCA: Bicinchoninic acid
BSA: Bovine serum albumin
NADH: Nicotinamide adenine dinucleotide (reduced)
NADPH: Nicotinamide adenine dinucleotide phosphate (reduced)
U: Enzymatic activity unit
MTT tetrazolium: 3-(4,5-Dimethylthiazol-2-yl)-2,5-diphenyltetrazolium
MTT formazan: 1-(4,5-Dimethylthiazol-2-yl)-3,5-diphenylformazan
HPLC: High performance liquid chromatography
TvQRD: *Trametes versicolor* 1,4-benzoquinone reductase
PcQRD: *Phanerochaete chrysosporium* 1,4-benzoquinone reductase
