# Supplementary material for: Heterologous expression of 1,4-benzoquinone reductases from white-rot fungi in Saccharomyces cerevisiae confers protection against p-benzoquinone toxicity
Source: Microb Cell Fact. 2026 Apr 8;25:102. doi: 10.1186/s12934-026-03001-1 (PMC13088443; doi:10.1186/s12934-026-03001-1)
Supplement: Supplementary file 1 — Supplementary Material 1: Fig. S1. Amino-acid sequence alignments of reported 1,4-benzoquinone reductases. Fig. S2. Zymogram of a native PAGE showing samples of intracellular medium from different constructs after growth (A600=4.0) in cultures supplemented with 10 mg L− 1 p-BQ. Fig. S3. SDS-PAGE gel showing intracellular extracts of different colonies from the empty vector control and full-length TvQRD. Table S1. Accession numbers of gene-level and protein-level sequences of QRDs included in the study. Table S2. Specific activity measurements. [file 12934_2026_3001_MOESM1_ESM.pdf]

Supplementary material of "Heterologous expression of 1,4-benzoquinone reductases from white-rot fungi in *Saccharomyces cerevisiae* confers protection against *p*-benzoquinone toxicity"

Mateo Bello Villarino[1] and Leif J. Jönsson[1*]

[1]Umeå University, Department of Chemistry, SE-901 87 Umeå, Sweden

a

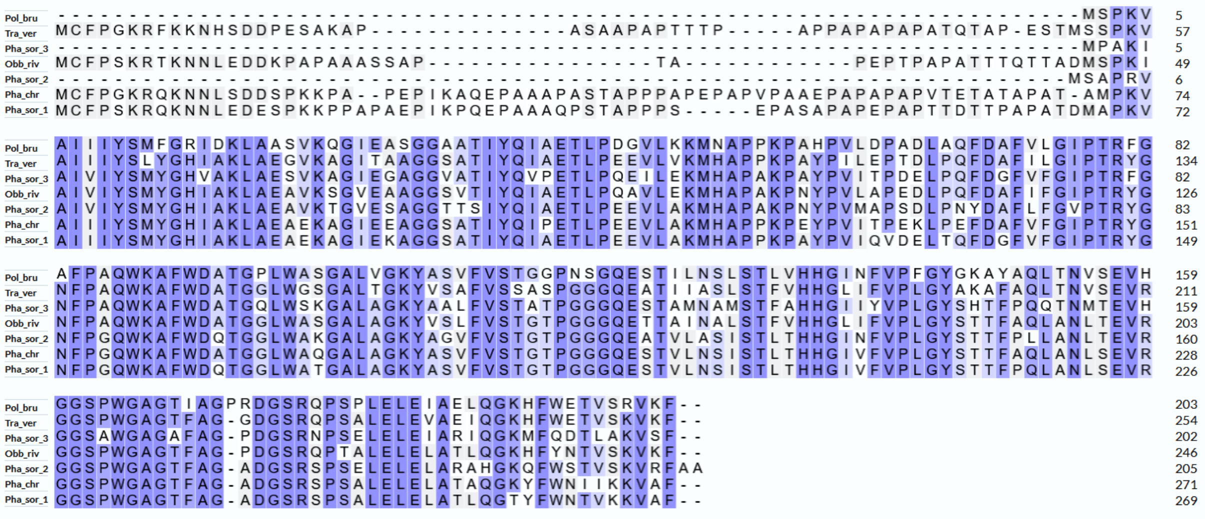

b

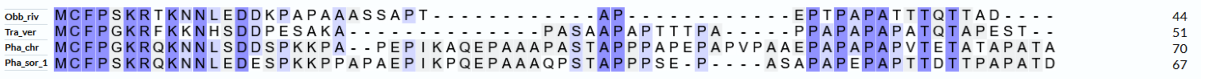

Fig. S1. Amino-acid sequence alignments of (a) reported 1,4-benzoquinone reductases in genomes of Polyporales fungi and (b) of hypothetical leader sequences in a selection of the sequences. Residue identities matching 50% highlighted in gray, above 50% highlighted in blue with hue increasing in intensity until 100% identity. Organisms included are *Polyporus brumalis* (Pol_bru), *Trametes versicolor* (Tra_ver), *Phanerochaete sordida* (Pha_sor) with three potential QRDs, *Obba rivulosa* (Obb_riv) and *Phanerochaete chrysosporium* (Pha_chr).

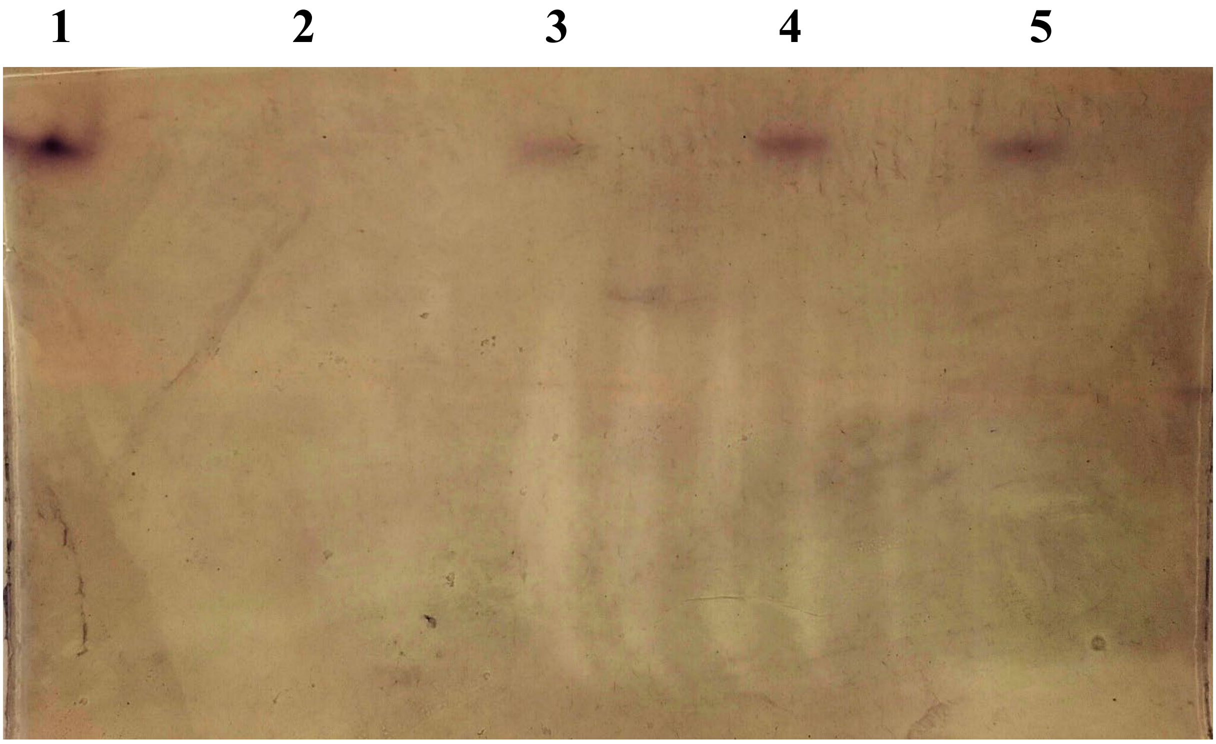

Fig. S2. Zymogram of a native PAGE showing samples of intracellular medium from different constructs after growth (A_{600}=4.0) in cultures supplemented with 10 mg L^{-1} *p*-BQ: Lane 1, full-length TvQRD; lane 2, empty vector control; lane 3, leaderless PcQRD; lane 4, leaderless TvQRD; lane 5, full-length PcQRD. The entire gel is shown, from which the activity-stained portion was cropped to become **Fig. 4**.

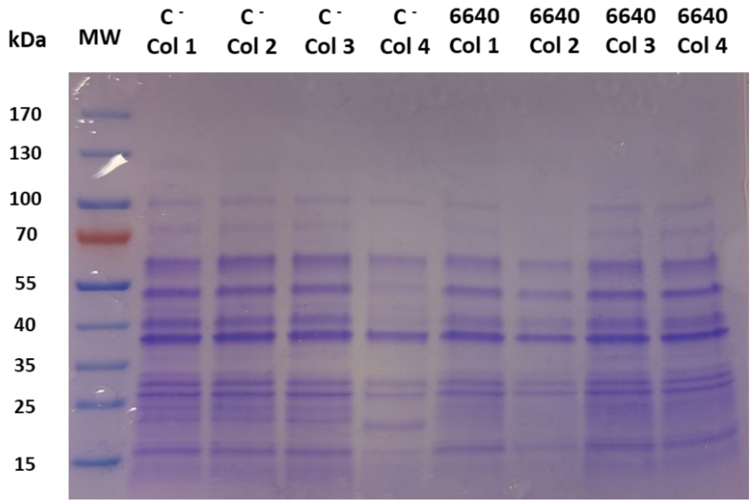

Fig. S3. SDS-PAGE gel showing the intracellular extracts of different colonies from the empty vector control (C^-) and full-length TvQRD (6640) constructs transformed into *S. cerevisiae* and cultured to A_{600}=4.0.

Table S1. Accession numbers of gene-level and protein-level sequences of QRDs included in the study. Gene accession numbers were obtained from GenBank and protein accession numbers were obtained from UniProt, with the exception of the sequence for *T. versicolor*, which was obtained from NCBI. Abbreviations include mRNA (messenger RNA), WGS (whole genome sequencing) and CDS (coding sequence).

Organism	Gene accession number	Protein accession number
Phanerochaete chrysosporium	AF106939.1 (mRNA)	Q9Y763
Obba rivulosa	KV722450.1 (WGS)	A0A8E2ASJ4
Polyporus brumalis	KZ857425.1 (WGS)	A0A371D2C8
Phanerochaete sordida 1	BPQB01000004.1 (WGS)	A0A9P3G1B2
Phanerochaete sordida 2	BPQB01000001.1 (WGS)	A0A9P3FXR4
Phanerochaete sordida 3	AB621802.1 (CDS)	F1T2K0
Trametes versicolor	JH711791.1 (WGS)	XP_008041687.1

Table S2. Specific activity measurements expressed in units of QRD activity (U) per mg of protein with NADH as reducing substrate. Values in parentheses are the standard deviation of triplicates.

Initial [*p*-BQ]	0 mg L^{-1}	2 mg L^{-1}	10 mg L^{-1}	20 mg L^{-1}
Empty vector control	0.004 (0.000)	0.022 (0.003)	0.011 (0.002)	N/A
Full-length TvQRD	0.077 (0.027)	0.079 (0.012)	0.141 (0.009)	0.242 (0.018)
Leaderless TvQRD	0.052 (0.009)	0.058 (0.017)	0.104 (0.012)	0.179 (0.021)
Full-length PcQRD	0.012 (0.007)	0.049 (0.004)	0.120 (0.008)	0.204 (0.027)
Leaderless PcQRD	0.010 (0.003)	0.025 (0.003)	0.086 (0.027)	0.123 (0.012)
